# An Overview of Biomolecular Event Extraction from Scientific Documents

**DOI:** 10.1155/2015/571381

**Published:** 2015-10-26

**Authors:** Jorge A. Vanegas, Sérgio Matos, Fabio González, José L. Oliveira

**Affiliations:** ^1^MindLab Research Laboratory, Universidad Nacional de Colombia, Bogotá, Colombia; ^2^DETI/IEETA, University of Aveiro, Campus Universitário de Santiago, 3810-193 Aveiro, Portugal

## Abstract

This paper presents a review of state-of-the-art approaches to automatic extraction of biomolecular events from scientific texts. Events involving biomolecules such as genes, transcription factors, or enzymes, for example, have a central role in biological processes and functions and provide valuable information for describing physiological and pathogenesis mechanisms. Event extraction from biomedical literature has a broad range of applications, including support for information retrieval, knowledge summarization, and information extraction and discovery. However, automatic event extraction is a challenging task due to the ambiguity and diversity of natural language and higher-level linguistic phenomena, such as speculations and negations, which occur in biological texts and can lead to misunderstanding or incorrect interpretation. Many strategies have been proposed in the last decade, originating from different research areas such as natural language processing, machine learning, and statistics. This review summarizes the most representative approaches in biomolecular event extraction and presents an analysis of the current state of the art and of commonly used methods, features, and tools. Finally, current research trends and future perspectives are also discussed.

## 1. Introduction

The scientific literature is the most important medium for disseminating new knowledge in the biomedical domain. Thanks to advances in computational and biological methods, the scale of research in this domain has changed remarkably, reflected in an exponential increase in the number of scientific publications [1]. This has made it harder than ever for scientists to find, manage, and exploit all relevant studies and results related to their research field [1]. Because of this, there is growing awareness that automated exploitation tools for this kind of literature are needed [2]. To address this need, natural language processing (NLP) and text mining (TM) techniques are rapidly becoming indispensable tools to support and facilitate biological analyses and the curation of biological databases. Furthermore, the development of this kind of tools has enabled the creation of a variety of applications, including domain-specific semantic search engines and tools to support the creation and annotation of pathways or for automatic population and enrichment of databases [3–5].

Initial efforts in biomedical TM focused on the fundamental tasks of detecting mentions of entities of interest and linking these entities to specific identifiers in reference knowledge bases [6, 7]. Although entity normalization remains an active research challenge, due to the high level of ambiguity in entity names, some existing tools offer performance levels that are sufficient for many information extraction applications [6]. In recent years there has been increased interest in the identification of interactions between biologically relevant entities, including, for instance, drug-drug [8] or protein-protein interactions (PPIs) [9]. Amongst these, the identification of PPIs mentioned in the literature has received most attention, encouraged by their importance in systems biology and by the necessity to accelerate the population of numerous PPI databases.

Following the advances achieved in PPI extraction, it became relevant to automatically extract more detailed descriptions of protein related events that depict protein characteristics and behavior under certain conditions. Such events, including expression, transcription, localization, binding, or regulation, among others, play a central role in the understanding of biological processes and functions and provide insight into physiological and pathogenesis mechanisms. Automatically creating structured representations of these textual descriptions allows their use in information retrieval and question answering systems, for constructing biological networks composed of such events [2] or for inferring new associations through knowledge discovery. Unfortunately, extraction of this kind of biological information is a challenging task due to several factors: firstly, the biological processes described are generally complex, involving multiple participants which may be individual entities such as genes or proteins, groups, or families, or even other biological processes; sentences describing these processes are long and in many cases have long-range dependencies; and, finally, biological text is also rich in higher level linguistic phenomena, such as speculation and negation, which may cause misinterpretation of the text if not handled properly [1, 9].

This review summarizes the different approaches used to address the extraction and formalization of biomolecular events described in scientific texts. The downstream impact of these advances, namely, for network extraction, for pharmacogenomics studies, and in systems biology and functional genomics, has been highlighted in recent reviews [2, 4, 10], which have also described various end-user systems developed on top of these technologies. This review focuses on the methodological aspects, describing the available resources and tools as well as the features, algorithms, and pipelines used to address this information extraction task, and specifically for protein related events, which have received the most attention in this perspective. We present and discuss the most representative methods currently available, describing the advantages, disadvantages, and specific characteristics of each strategy. The most promising directions for future research in this area are also discussed.

The contents of this paper are organized as follows: we start by introducing biomolecular events and defining the event extraction task; we then describe the event extraction steps, present commonly used frameworks, text processing, and NLP tools and resources, and compare the different approaches used to address this task; in the following section we compare the performance of the proposed methods and systems, followed by a discussion regarding the most relevant aspects; finally, we present some concluding remarks in the last section.

## 2. Biomolecular Events

In the biomedical domain, an event refers to the change of state of one or more biomedical entities, such as proteins, cells, and chemicals [11]. In their textual description, an event is typically referenced through a trigger expression that specifies the event and indicates its type. These triggers are generally verbal forms (e.g., “stimulates”) or nominalizations of verbs (e.g., “expression”) and may occur as a single word or as a sequence of words. This textual description also includes the entities involved in the event, referred to as participants, and possibly additional information that further specifies the event, such as a particular cell type in which the described event was observed. Biomolecular events may describe the change of a single gene or protein, therefore having only one participant denoting the affected entity, or may have multiple participants, such as the biomolecules involved in a binding process, for example. Additionally, an event may act as participant in a more complex event, as in the case of regulation events, requiring the detection of recursive structures.

Extraction of event descriptions from scientific texts has attracted substantial attention in the last decade, namely, for those events involving proteins and other biomolecules. This task requires the determination of the semantic types of the events, identifying the event participants, which may be entities (e.g., proteins) or other events, their corresponding semantic role in the event, and finally the encoding of this information using a particular formalism. This structured definition of events is associated with an ontology that defines the types of events and entities, semantic roles, and also any other attributes that may be assigned to an event. Examples of ontologies for describing biomolecular events include the GENIA Event Ontology [11] and Gene Ontology [12].

**Figure 1:**
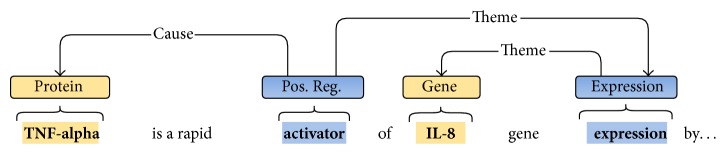
Example of complex biomolecular event extracted from a text fragment. A recursive structure, composed of two types of events, is presented: Positive Regulation and Expression.

Figure 1 presents an example of a complex event described in the text fragment “*TNF-alpha is a rapid activator of IL-8 gene expression by*….” From this fragment we can construct a recursive structure composed of two events: a first event, of type* Expression* denoted by the trigger word “*expression*” that has a single argument (“IL-8”) with the role* Theme* (denoting that this is the participant affected by the event), and a second event of type* Positive Regulation*, defined by the trigger word “*activator.*” This second event has two participants: the protein “TNF-alpha” with the role* Cause* (defining that this protein is the cause of the event) and the first event with the role* Theme*.

## 3. Event Extraction

**Figure 2:**
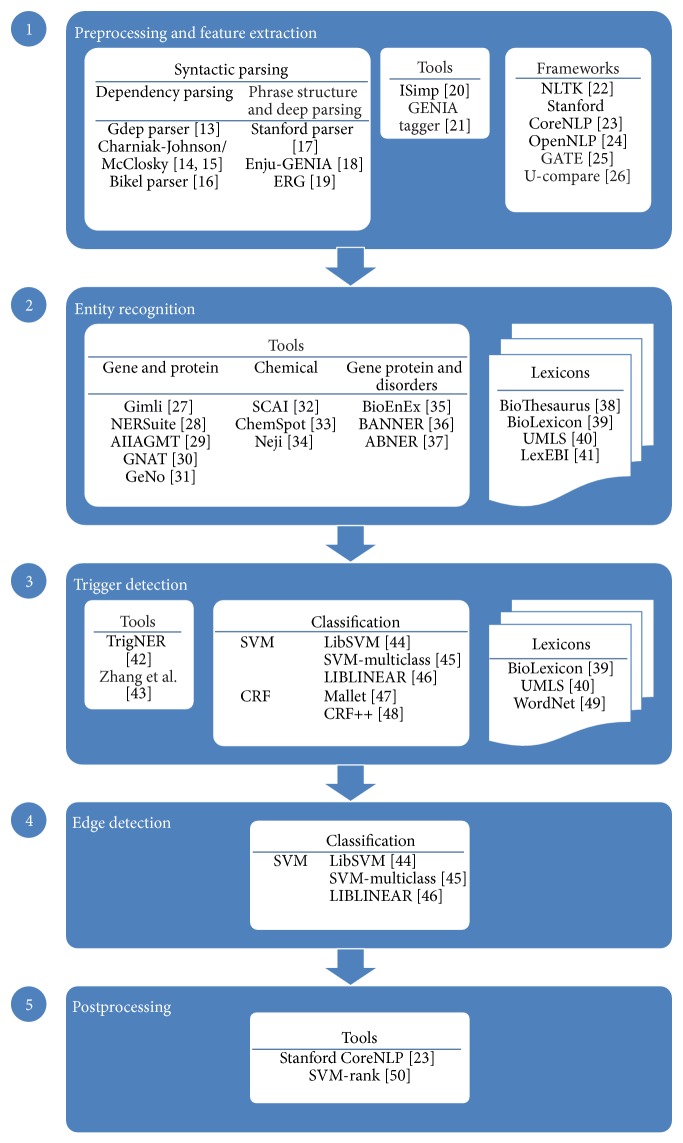
Overall pipeline of a biomedical event extraction solution. Joint prediction methods merge steps 3 and 4 in a single step. The corresponding reference paper for each tool and method is also identified [13–50].

Figure 2 illustrates a common event extraction pipeline, identifying the most popular tools, models, and resources used in each stage. The two initial stages are usually preprocessing and feature extraction, followed by the identification of named entities. The next step is to perform event detection. This step is frequently divided into two separate stages: trigger detection, which consists of the identification of event triggers and their type, and edge detection (or event construction), which is focused on associating event triggers with their arguments. Some authors, on the other hand, have addressed event detection in a single, joint prediction step. These approaches tackle the cascading errors that occur with the two-stage methods and have commonly shown improved performance. Finally, a postprocessing stage is usually present, to refine and complete the candidate event structures. Negation or speculation detection may also be included in this final step. This section describes each phase, presenting the most commonly used approaches.

### 3.1. Corpora for Event Extraction

The development and improvement of information extraction systems usually requires the existence of manually annotated text collections, or corpora. This is mostly true for supervised machine learning methods, but annotated data can also be exploited for inferring patterns to be used in rule-based approaches. In the case of biomedical event extraction, various corpora have been compiled, including corpora annotated with protein-protein interactions.

#### 3.1.1. GENIA Event Corpus

The GENIA Event corpus contains human-curated annotations of complex, nested, and typed event relations [51, 52]. The GENIA corpus [53] is composed of 1,000 paper abstracts from Medline. It contains 9,372 sentences from which 36,114 events are identified. This corpus is provided by the organizers of BioNLP shared task to participants as the main resource for training and evaluation and is publicly available online (http://www.nactem.ac.uk/aNT/genia.html).

#### 3.1.2. BioInfer Corpus

BioInfer (Biomedical Information Extraction Resource) (http://www.it.utu.fi/BioInfer) [54] is a public resource providing manually annotated corpus and related resources for information extraction in the biomedical domain.

The corpus contains sentences from abstracts of biomedical research articles annotated for relationships, named entities, and syntactic dependencies. The corpus is annotated with proteins, genes, and RNA relationships and serves as a resource for the development of information extraction systems and their components such as parsers and domain analyzers. The corpus is composed of 1100 sentences from abstracts of biomedical research articles.

#### 3.1.3. Gene Regulation Event Corpus

The Gene Regulation Event Corpus (GREC) (http://www.nactem.ac.uk/GREC/) [55] consists of 240 MEDLINE abstracts, in which events relating to gene regulation and expression have been annotated by biologists. This corpus has the particularity that not only core relations between entities that are annotated, but also a range of other important details about these relationships, for example, location, temporal, manner, and environmental conditions.

#### 3.1.4. GeneReg Corpus

The GeneReg Corpus [56] consists of 314 MEDLINE abstracts containing 1770 pairwise relations denoting gene expression regulation events in the model organism* E*.* coli*. The corpus annotation is compatible with the GENIA event corpus and with in-domain and out-of-domain lexical resources.

#### 3.1.5. PPI Corpora

Although not as richly annotated as event corpora, protein-protein interaction corpora may be considered for complementing the available training data. The most relevant PPI corpora are the LLL corpus [57], the AIMed corpus [58], and the BioCreative PPI corpus [7].

### 3.2. Preprocessing and Feature Extraction

Preprocessing is a required step in any text mining pipeline. This includes reading the data from its original format to an internal representation, and extracting features, which usually involves some level of text or language processing. In the specific case of event extraction, preprocessing may also involve resolving coreferences [59] or applying some form of sentence simplification [60], for example, by expanding conjunctions, in order to improve the extraction results.

#### 3.2.1. Preprocessing Tools

*Frameworks*. In order to derive a feature representation from texts, it is necessary to perform text processing involving a set of common NLP tasks, going from sentence segmentation and tokenization, to part-of-speech tagging, chunking, and linguistic parsing. Various text processing frameworks exist that support these tasks, among which the following stand out: NLTK (http://www.nltk.org/), Apache OpenNLP (https://opennlp.apache.org/), and Stanford CoreNLP (http://nlp.stanford.edu/software/corenlp.shtml) (Figure 2).

*Syntactic Parsers*. A syntactic parser assigns a tree or graph structure to a free text sentence. These structures establish relations or dependencies between the organizing verb and its dependent arguments and have been useful for many applications like negation detection and disambiguation among others. Syntactic parsers can be categorized in three groups: dependency parsers, phase structure parsers, and deep parsers [61]. The aim of dependency parsers is to compute a tree structure of a sentence where nodes are words, and edges represent the relations among words; phrase structure parsers focus on identifying phrases and their recursive structure, and deep parsers express deeper relations by computing theory-specific syntactic/semantic structures. For the task of event extraction several implementations of each parser groups have been used, as shown in Figure 2.

#### 3.2.2. Features

One of the main requirements of a good event extraction system is a rich feature representation. Most event extraction systems present a complex set of features extracted from tokens, sentences, dependency parsing trees, and external resources.  Table 1 summarizes the features commonly extracted in this processing stage and indicates their use in the event extraction process.

- Token-based features capture specific knowledge regarding each token, such as syntactic or linguistic features, namely, part-of-speech (POS) and the lemma of each token, and features based on orthographic (e.g., presence of capitalization, punctuation, and numeric or special characters) [42, 43, 62–68] and morphological information, namely, prefixes, suffixes, and character n-grams [42, 43, 64, 67, 69–72].
- Contextual features provide general characteristics of the sentence or neighborhood where the target token is present. Features extracted from sentences include the number of tokens in the sentence [42], the number of named entities in the sentence, and bag-of-word counts of all words [43, 64]. Local context is usually encoded through windows or conjunctions of features, including POS tags, lemmas, and word n-grams, extracted from the words around the target token [42, 63, 65, 73].
- Dependency parsing provides information about grammatical relationships involving two words, extracted from a graph representation of the dependency relations in a sentence. Commonly used features include the number or type of dependency hops between two tokens, and the sequence or n-grams of words, lemmas, or POS tags in the dependency path between two tokens [65, 68, 72, 74]. These features are usually extracted between two entities in a sentence [64, 75], or between a candidate trigger and an entity [75].
- Finally, it is also common to encode domain knowledge as features using external resources such as lexicons of possible trigger words and of gene and protein names to indicate the presence of a candidate trigger or entity [27, 76–78]. Also, the token representation is often expanded with related words according to some semantic relations such as WordNet hypernyms [27, 77, 79].

### 3.3. Entity Recognition

Entity recognition consists of the detection of references (or mentions) to entities, such as genes or proteins, in natural language text and labeling them with their location and type. Named-entity recognition in the biomedical domain is generally considered to be more difficult than in other domains, for several reasons: first, there are millions of entity names in use [71] and new ones are added constantly, implying that dictionaries cannot be sufficiently comprehensive; second, the biomedical field is evolving too quickly to allow reaching a consensus on the name to be used for a given entity [80] or even regarding the exact concept defined by the entity itself. So the same name or acronym can be used for different concepts [81].

Several entity recognition systems for the biomedical domain have been developed in the last decade. Much of this work has focused on the recognition of gene and protein names and, more recently, chemical compounds [82]. In these cases, machine learning strategies using rich sets of features have provided the best results, with performances in the order of 85%  *F*-measure [83].

The most popular entity recognition tools are shown in Figure 2, which also lists the biomedical lexicons that are commonly used, either in dictionary-matching approaches or as features for machine learning. Some of these tools, namely, BANNER [36] and Gimli [27], offer simple interfaces for training new models and have been applied to the recognition of various entity types such as chemical compounds and diseases.

### 3.4. Trigger Detection

Trigger word detection is the event extraction task that has attracted most research interest. It is a crucial task, since the effectiveness of the following tasks strongly depends on the information generated in this step. This task consists of identifying the chunk of text that triggers the event and serves as predicate. Although trigger words are not restricted to a particular set of part-of-speech tags, verbs (e.g., “activates”) and nouns (e.g., “expression”) are the most common. Furthermore, a trigger may consist of multiple consecutive words.

**Figure 3:**
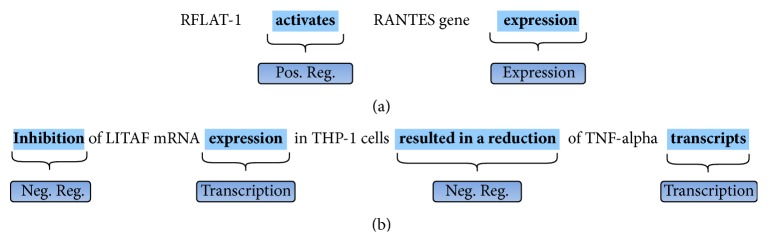
Trigger detection for two example sentences: (a) “RFLAT-1 activates RANTES gene expression” and (b) “Inhibition of LITAF mRNA expression in THP-1 cells resulted in a reduction of TNF-alpha transcripts.”

Figure 3 illustrates the expected results of the trigger detection process in two example sentences. As we can see in Figure 3, trigger detection involves the identification of event triggers and their type, as specified by the selected ontology. In sentence (a), two different kinds of events are identified: the trigger word* activates* defines an event of type* Positive Regulation* and the trigger word* expression* defines an event of type* Gene Expression*. Sentence (b) illustrates the difficulty of this task: it shows that short sentences can contain various related events; that triggers may be expressed in diverse ways (two event of type* Negative Regulation* are defined with different trigger words); and, finally, that the same trigger word (*expression*) may indicate different types of event, depending on the context.

The various approaches proposed for trigger detection can be roughly categorized in three types: rule-based, dictionary-based, and machine learning-based. These approaches are summarized in Table 2 and presented in the remainder of this section.

#### 3.4.1. Patterns and Matching Rules for Trigger Detection

There are several strategies based on patterns [70, 93] and matching rules. Rule-based methods commonly follow some manually defined linguistic patterns, which are then augmented with additional constraints based on word forms and syntactic categories to generate better matching precision. The main advantage of this kind of approach is that they usually require little computational effort. Rule-based event extraction systems consist of a set of rules that are manually defined or generated from training data. For instance, Casillas et al. [88] present a strategy based on Kybots (Knowledge Yielding Robots), which are abstract patterns that detect actual concept instances and relations in a document. These patterns are defined in a declarative format, which allows definition of variables, relations, and events. Vlachos et al. [76] present a domain-independent approach based on the output of a syntactic parser and standard linguistic processing (namely, stemming, lemmatization, and part-of-speech (POS) tagging, among others), augmented by rules acquired from the development data in an unsupervised way, avoiding the need to use explicitly annotated training data.

In the dictionary-based approach, a dictionary containing trigger words with their corresponding classes (event types) is used to identify and assign event triggers. Van Landeghem et al. [74] proposed a strategy following this approach, using a set of manually cleaned dictionaries and a formula to calculate the importance of each trigger word for a particular event. This is required since the same word may be associated with events of different types [66]. For instance, in the BioNLP'09 Shared Task dataset [51], the token “overexpression” appears as trigger for the gene expression event in about 30% of its occurrences, while the other 70% of occurrences are triggers for positive or negative regulation events.

Many strategies combine both approaches. For instance, Le Minh et al. [70] present a strategy where rule-based and dictionary-based approaches are combined. First, they select tokens that have appropriate POS tags and occur near a protein mention and then apply heuristic rules extracted from a training corpus to identify candidate triggers. Finally, a dictionary built from the training corpus and containing trigger words and their corresponding classes is used to classify candidate triggers. For ambiguous trigger classes, the class with the highest rate of occurrence is selected. Kilicoglu and Bergler [93] also present a combined strategy based on a linguistically inspired rule-based and syntax-driven methodology, using a dictionary based on trigger expressions collected from the training corpus. Events are then fully specified through syntactic dependency based heuristics, starting from the triggers detected by the dictionary-matching step.

Pattern-based methods usually present low recall rates, since defining comprehensive patterns would require extensive efforts, and because the most common patterns are too rigid to capture semantic/syntactic paraphrases.

#### 3.4.2. Machine Learning-Based Approach to Trigger Detection

The most recent and successful approaches to trigger word detection are based on machine learning methods [72], with most work defining this as a sequence-labeling problem. The definition of event types, on the other hand, is addressed as a multiclass task, where candidate event triggers are classified into one of the predefined types of biomedical events. In order to address these problems, several probabilistic techniques have been proposed, using, for example, Hidden Markov Models (HMMs), Maximum Entropy Markov Models (MEMMs), Conditional Random Fields (CRFs) [94, 95], and Support Vector Machines (SVMs).

For instance, Zhou and He [89] proposed treating trigger identification as a sequence-labeling problem and use the Maximum Entropy Markov Model (MEMM) to detect trigger words. MEMM is based on the concept of a probabilistic finite state model such as HMM but consists of a discriminative model that assumes the unknown values to be learnt are connected in a Markov chain rather than being conditionally independent of each other. Similarly, various strategies based on Conditional Random Fields (CRFs) have been proposed [42, 73, 85, 86]. CRFs have become a popular method for sequence-labeling problems, justified mainly by the fact that CRFs avoid the label bias problem present in MEMMs [96] but preserve all the other advantages. Unlike Hidden Markov Models (HMMs), CRF is a discriminant model. So CRFs use conditional probability for inference, meaning that they maximize *p*(*y*∣*x*) directly, where *x* is the input sequence and *y* is the sequence of output labels, unlike HMMs, which maximize the joint probability *p*(*x*, *y*). This relaxes strong independence assumptions required to learn the parameters of generative models.

The most recent proposals for trigger detection are based on Support Vector Machines (SVMs). SVMs do not follow a probabilistic approach but are instead maximum margin classifiers that try to find the maximal separation between classes. This classifier has presented very good results, showing a higher generalization performance than CRFs. However, training complex SVM models may require excessive computational time and memory overhead. Several strategies using different SVM implementations and kernels have been proposed.

The general approach is to classify initial candidate triggers as positive or not, based on a set of carefully selected features and a training set with annotated events. For instance, Björne et al. [80, 86, 97] proposed a solution based on the SVM-multiclass (http://www.cs.cornell.edu/people/tj/svm_light/svm_multiclass.html) implementation with a linear kernel, optimized by exploring in an exhaustive grid search the *C*-parameter that maximizes the *F*-score in trigger detection. In this study only linear kernels were used since the size and complexity of the training set, composed of over 30 thousand instances and nearly 300 thousand features, hinders the application of more computationally demanding alternatives, namely, radial basis function kernels.

In addition to purely supervised learning, which depends on the amount and quality of annotated data, semisupervised approaches have also been proposed. Wang et al. [65] combined labeled data with large amounts of unlabeled data, using a rich representation based on semantic features (such as walk subsequence features and n-gram features, among others) and a new representation based on Event Feature Coupling Generalization (EFCG). EFCG is a strategy to produce higher-level features based on two kinds of original features: class-distinguishing features (CDFs) which have the ability to distinguish the different classes and example-distinguishing features (EDFs) that are good at indicating the specific examples. EFCG generates a new set of features by combining these two kinds of features and taking into account a degree of relatedness between them.

A different strategy was followed by Martinez et al., who presented a solution based on word-sense disambiguation (WSD) using a combined CRF-VSM (Vector Space Model) classifier, where the output of VSM is incorporated as a feature into the CRF [73]. This approach significantly improved the performance of each method separately.

### 3.5. Edge Detection

**Figure 4:**
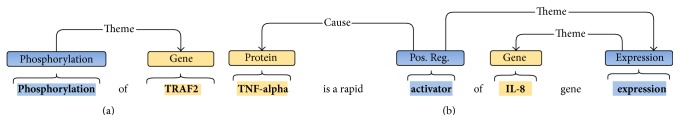
Event extraction from two example sentences: (a) “phosphorylation of TRAF2” and (b) “TNF-alpha which is a rapid activator of IL-8 gene expression.”

Edge detection (also known as event theme construction or event argument identification) is the task of predicting arguments for an event, which may be named entities (i.e., genes and proteins) or another event, represented by another trigger word. Event arguments are graphically represented through directed edges from the trigger word for the event and the argument. These edges also express the semantic role that a participant (entity or event) plays in a given event. In Figure 4, sentence (a) illustrates a basic event defined by the trigger word* Phosphorylation* that denotes an event of type* Phosphorylation*. The directed edge between this trigger word and the entity* TRAF2*, denoting a relation of type “Theme,” indicates that this entity is the affected participant in this event. It is important to note that events can act as participants in other events, thus allowing the construction of complex conceptual structures. For example, consider the sentence (c), where two events are mentioned: a first event of type* Expression* and a second event of type* Positive Regulation*. The directed edge from the trigger word* activator* and the trigger word* expression* denotes that the event* Expression* is affected directly by the event* Positive Regulation*. Similarly, the edge of type cause between* activator* and the entity* TNFalpha* indicates that this is the causing participant for this event.

Different approaches have been suggested to tackle the edge detection task, including rule and dictionary-based strategies and machine learning-based methods. These are summarized in Table 3 and described in the following subsections.

#### 3.5.1. Patterns and Matching Rules for Edge Detection

These strategies are based on the identification of edges according to a set of rules that can be manually defined or generated from training data. Among the most basic approaches, we find the strategy proposed by MacKinlay et al. [85], in which a specific set of hand-coded grammars, supported by specific domain knowledge like named entity annotations and lexicons, is defined for each type of event. In the case of basic events a simple distance criterion is applied, assigning the closest protein as the theme of the event, while extra criteria is required for more complex events. For instance, to assign the* Theme* arguments for binding events, the maximum distance away from the trigger event word(s), and the maximum number of possible themes are estimated, and for regulation events, in addition to the maximum distance, some priority rules are used to define* Cause* or* Theme* arguments.

Kilicoglu and Bergler [93] present another rule-based approach, where identification of the event participants and corresponding roles (e.g.,* Theme* or* Cause*) is primarily achieved based on a grammar created from dependency relations between event trigger expressions and event arguments in the training corpus. This strategy is based on the Stanford syntactic parser [98], which was applied to automatically extract dependency relation paths between event triggers and their corresponding event arguments. These paths were manually filtered, preserving only the correct and sufficiently general ones.

Le Minh et al. [70] follow a similar strategy by generating pattern lists from training data using the dependency graphs resulting from application of a deep syntactic parser.

Bui et al. [99] present one of the most recent studies based on dictionaries and patterns automatically generated from a training set. In this work, less than one minute was required to process a training set composed of about 950 abstracts on a computer with 4 gigabytes of memory, illustrating a main advantage of rule-based systems. Unfortunately, despite the low computational requirements, this kind of approach usually shows modest performance in terms of recall, due to the difficulty in modeling more complex relationships and in defining rules capable of generalizing.

#### 3.5.2. Machine Learning-Based Approach to Edge Detection

In recent years, similarly to trigger detection, there has been a clear tendency to approach the edge detection task using machine learning methods. Most works agree on addressing this problem as a supervised multiclass classification problem by defining a limited number of edge classes.

As can be seen in Table 3, most approaches are based on SVMs. Miwa et al. [87] presented one such approach, dividing the task into two different classification problems: edge detection between two triggers and edge detection between a trigger and a protein. For this purpose a set of annotated instances is constructed from a training set, as follows: for each event found in the training set, a list of annotated edges is constructed using as label the combination of the corresponding event class and the edge type (e.g., Binding: Theme). Using these extracted annotated edges, an unbalanced classification problem is then solved using one-versus-rest linear SVMs. Björne et al. [64] and Wang et al. [65] followed similar approaches, using multiclass SVMs in which two kinds of edges are annotated: trigger-trigger and trigger-protein. Each example is classified as* Theme*,* Cause,* or* Negative* denoting the absence of an edge between the two nodes. Each edge is predicted independently, so that the classification is not affected by positive or negative classification of other edges.

Roller and Stevenson [68] evaluated a similar strategy, using a polynomial kernel. The classification of the relations is carried out in three stages. The first consists of the identification of basic events by defining the trigger and a theme referring to a protein; the second stage seeks to identify regulation events by defining the trigger and a theme referring to a trigger from a previously identified basic event; and the final stage tries to identify additional arguments. Hakala et al. [91] proposed a reranking approach that uses the prediction scores of a first SVM classifier and information about the event structure as inputs for a new SVM model focused on optimizing the ranking of the predicted edges. For this new model, polynomial and radial basis kernels were evaluated, showing an improvement in the overall precision of the system.

A different strategy was used by Zhou and He [89], who proposed a method based on a Hidden Vector State model, called HVS-BioEvent. Although this method presented lower performance in basic events, compared to systems based on SVM classifiers, it achieved better performance in complex events due to the hierarchical hidden state structure. This structure is indeed more suitable for complex event extraction since it can naturally model embedded structural context in sentences.

Van Landeghem et al. [74] proposed an approach that processes each type of event in parallel using binary SVMs. All predictions are assembled in an integrated graph, on which heuristic postprocessing techniques are applied to ensure global consistency. Linear and radial base function (RBF) kernels were evaluated by performing parameter tuning via 5-fold cross-validation. Van Landeghem et al. made an interesting exploration about feature selection; they applied fully automated feature selection techniques aimed at identifying a subset of the most relevant features from a large initial set of features. An analysis of the results showed that up to 50% of all features can be removed without losing more than one percentage point in *F*-score, while at the same time creating faster classification models.

#### 3.5.3. Hybrid Approaches

In the literature, we can find many studies that combine ML-based with rule-based and dictionary-based strategies. This combination is often performed in two ways: (1) in an ensemble strategy, each method is performed independently and the final output is obtained by combining the results of each method, either through rules or by using some classification or regression model; and (2) in a stacked strategy, the output of one method is used as input for the following one that performs a filtering and refining process to produce a more accurate final output.

As an example of the first kind of approach, Pham et al. [100] proposed a hybrid system that combines both rule-based and machine learning-based approaches. In this method, the final list of predicted events is given by the combination of the events extracted by rule-based methods based on syntactic and dependency graphs and those extracted via SVM classifiers. In the second kind of approach, several studies [68, 80, 97] have used a rule-based postprocessing step to refine the initial resulting graph generated by ML-based classifiers by eliminating duplicate nodes and separating their edges into valid combinations based on the syntax of the sentences and the conditions in argument type combinations, taking into account the characteristics and peculiarities of each kind of event.

#### 3.5.4. Structured Prediction and Joint Models

To address the potential cascading errors that originate from two-stage approaches described above, some authors have proposed the joint prediction of triggers, event participants, and connecting edges. Riedel et al. [101] and Poon and Vanderwende [102] proposed two methods based on Markov logic. Markov logic is an extension to first-order logic in which a probabilistic weight is attached to each clause [103]. Instead of using the relational structures over event entities, as represented in Figure 4, Riedel et al. represent these as labeled links between tokens of the sentence and apply link prediction over token sequences. As stated by the authors, this link-based representation simplifies the design of the Markov Logic Network (MLN). Poon and Vanderwendle, on the other hand, used Markov logic to model the dependency edges obtained with the Stanford dependency parser. The resulting MLN therefore jointly predicts if a token is a trigger word, the corresponding event type, and which of the token's dependency edges connect to (Theme or Cause) event arguments. This allows using a simpler set of features in the MLN, which leads to a more computationally efficient solution without sacrificing the prediction performance. The authors used heuristics to fix two typical parsing errors, namely, propositional phrase attachment and coordination, and showed that this had an important impact on the final results.

Riedel and McCallum [104] proposed another approach in which the problem is decomposed in three submodels: one for extracting event triggers and outgoing edges, one for event triggers and incoming edges, and one for protein-protein bindings. The optimization methods for the three submodels are combined via dual decomposition [105], with three types of constraints enforced to achieve a joint prediction model. Links between tokens are represented through a set of binary variables as in Riedel et al. [101].

McClosky et al. [98] proposed a different approach, in which event structures are converted into dependencies between event triggers and event participants. Various dependency parsers are trained using features from these dependency trees as well as features extracted from the original sentences. In recognition phase, the parsing results are converted back to event structures and ranked by a maximum-entropy reranker component.

Vlachos and Craven [106] applied the search-based structured prediction framework (SEARN) to the problem of event extraction. This approach decomposes event extraction into jointly learning classifiers for a set of classification tasks, in which each model can incorporate features that represent the predictions made by the other ones. Moreover, the loss function incorporates all predictions, which means that the models are jointly learned and a structured prediction is achieved. For this specific task, models were trained to classify each token as a trigger or not and to classify each possible pair of trigger-theme and trigger-cause in a sentence.

### 3.6. Modality Detection

Modality detection refers to the crucial part of identifying negations and speculations [107]. The aim of this task is to avoid opposite meanings and to distinguish when a sentence can be interpreted as subjective or as a nonfactual statement. The detection of speculations (also referred to as hedging) in the biomedical literature has been the focus of several recent studies, since the ability to distinguish between factual and uncertain information is of vital importance for any information extraction task [108].

In many approaches, modality detection is addressed as an extra phase following the edge detection process. Most approaches address this problem in two steps: first speculation/negation cues (which may be words such as “may,” “might,” “suggest,” “suspect,” and “seem,”) are detected, and, next, the scope of the cues is analyzed. Most of the initial systems were rule-based and relied on lexical or syntactic information, but recent studies have looked at solving this problem using binary classifiers [64, 78, 85] trained with generated instances annotated as negation, speculation, or negative (see Table 4).

## 4. Comparison of Existing Methods

In this section we present a comparative analysis of the different approaches and systems described in this review. To achieve a consistent comparison, we use the results achieved by the different systems on the standard datasets from the BioNLP shared tasks on event extraction [51, 52, 109]. These datasets provide a direct point of comparison and are commonly used to validate and evaluate new approaches and development, which endorses their use in this comparative analysis. The datasets are based on the GENIA corpus [53], consisting of a training set with 800 abstracts and a development set with 150 abstracts. The test data, composed of 260 abstracts, comes from an unpublished portion of the corpus. For the second edition of the challenge, this initial dataset was extended with 15 full-text articles, equally divided into training, development, and test portions. Evaluation is performed with standard recall, precision, and *F*-score metrics.

### 4.1. BioNLP Shared Task on Event Extraction

The BioNLP shared task series is the main community-wide effort to address the problem of event extraction, providing a standardized dataset and evaluation setting to compare and verify the evolution in performance of different methods. Since its initial organization in 2009, the BioNLP-ST series has defined a number of fine-grained information extraction (IE) tasks motivated by bioinformatics projects. In this analysis, we focus on the main task, GENIA Event Extraction (GE). This task focuses on the recognition of biomolecular events defined in the GENIA Event Ontology, from scientific abstracts or full papers. From the first edition three separate subtasks have been defined, each addressing the event extraction with a different level of specificity.

*Task 1*. Core event extraction: it consists of the identification of trigger words, associated with 9 events related to protein biology. The annotation of protein occurrences in the text, used as arguments for event triggers, is provided in both the training and the test sets.

*Task 2*. Event enrichment: it is recognition of secondary arguments that further specify the events extracted in Task 1. 

*Task 3*. Negation/speculation detection: it is detection of negations and speculation statements concerning extracted events.

#### 4.1.1. Target Event Types

The shared task defined a subset of nine biomolecular events from the GENIA Event Ontology, classified in three kinds with different levels of complexity: basic events, binding events, and regulation events. Basic events are the simplest to fully resolve, because these only require the specification of a primary argument. Five types of events are categorized in this group:* gene expression*,* transcription*,* protein catabolism*,* phosphorylation,* and* localization*.* Binding* events, on the other hand, require the detection of at least two arguments. Finally,* regulation* events, including* Negative* and* Positive Regulation*, are the most difficult to fully specify, because these involve the definition of another argument, which may be an entity or another event, requiring identification of a recursive structure.

### 4.2. Comparative Analysis

#### 4.2.1. Core Event Extraction

Table 5 summarizes the performance achieved by the most representative strategies addressing the core event extraction subtask (Task 1). The best results achieved during the first edition of the BioNLP-ST were obtained through machine learning techniques, formulating the problems of trigger and edge detection as different multiclass classification problems, solved by using linear SVM classifiers [86]. Using the same approach, Miwa et al. [87] reported improvements over these results by adding a set of shortest path features between triggers and proteins for the edge detection problem. As can be observed from the table, a considerable improvement was obtained for binding events, with an increase of over 12 percentage points in recall and 3 points in precision.

In BioNLP-ST 2011, the datasets were extended to include full text articles, but the abstract collection used for the first edition was maintained in order to measure the progress between the two editions. The best result in the second edition, an *F*-score of 57.46% when considering only the abstracts, was obtained by the FAUST system. This corresponds to a substantial increase of more than four percentage points over the previous best system, resulting from an improvement in the recognition of simple events but especially from a much better recognition of complex regulation events, with an increase of over 11 percentage points in precision and 3 points in recall.

The FAUST system consists of a stacked combination of two models: the Stanford event parser [98] was used for constructing dependency trees that were then used as additional input features for the second model, the UMass model [104]. The main distinction of the UMass model is that it performs joint prediction of triggers, arguments, and event structures, therefore overcoming the cascading errors that occur in the common pipeline approaches when, for example, a trigger is not correctly predicted in the first stage [111]. In this model, the problem of event extraction is divided into smaller simple subproblems that are solved individually, with each subproblem presenting a set of penalties that are added to an objective function. The final solution is found via an iterative tuning of the penalties until all individual solutions are consistent with each other. When used separately, the UMass model achieved the second best-performing results in this edition and was the top performing system when considering just full-texts. In its third edition, BioNLP-ST focused on simulating a more realistic scenario. For this reason, a new dataset was constructed using only recent full papers, so that the extracted information could represent up-to-date knowledge of the domain. Unfortunately, the collection of abstracts used in the first two editions (BioNLP-ST 2009 and BioNLP-ST 2011) was removed from the official evaluation and the full text collection used in the 2011 edition corresponds only to a small part of dataset used in this edition, making it difficult to compare against previous results and measure the progress of the community.

In this latest edition of the shared task the best-performing systems were EVEX [91] and TEES [97]. TEES, an evolution of the UTurku system and also mainly based on SVM classifiers, introduces an automated annotation scheme learning system that derives task-specific event rules and constraints from the training data. In turn, EVEX is a combined system that takes the outputs predicted by TEES and tries to reduce false positives by applying a reranking that assigns a numerical score to events and removing all events that are below a defined threshold. For this reranking, SVM^rank^ is used with a set of features based on confidence scores (i.e., maximum/minimum trigger confidence and maximum/minimum argument confidence, among others) and features describing the structure of the event (i.e., event type of the root trigger and paths in the event from root to arguments, among others). This reranking and filtering approach provided a small overall improvement, achieved through a better precision in the definition of regulation events, which constitute a substantial fraction of the annotated data [105].

BioSEM [99], a rule-based system based on patterns automatically derived from annotated events also achieved high performance results, with only marginal differences to the machine learning approaches described above. BioSEM learns patterns of relations between an event trigger and its arguments defined at three different levels: chunk, phrase, and clause. Notably, this system presents significantly greater precision than ML-based systems, especially considering simple and binding events with improvements of more than seven percentage points. While in the case of simple events this was accompanied by a decrease in recall, for binding events this rule-based system achieved the best results with a difference of over six percent in *F*-score. These results indicate that although ML methods still produce the best generalization, rule-based systems can approximate those results with much better precision and further suggests the combination of the two approaches.

#### 4.2.2. Event Enrichment

Table 6 shows the results obtained in the BioNLP-ST Task 2, which consists of the recognition of secondary event arguments. These secondary arguments depend on the type of event and include* Location* arguments (i.e.,* AtLoc* or* ToLoc*) that define the source or destination of an event and* Site* arguments (i.e.,* Site* or* Csite*) that indicate domains or regions to better specify the Theme or Cause of an event. The settings of this subtask changed between editions, not only in terms of the dataset used, but also in terms of the sites to be predicted as secondary arguments. This means that the results shown in the table are not directly comparable, namely, for the last edition of the challenge in which sites for different protein modification and regulation events were also considered. Nevertheless, these results were included for reference.

Considering the analysis of abstracts, the table shows an evident improvement on the results achieved by the top performing systems in the first and second editions. More interestingly, there is a considerable difference between the results achieved over full-texts and the results obtained on abstracts. This is an indication that, as expected, the language used for describing the events is much more complex in the main body of the articles, where events are specified in more detail, than in the abstracts. Moreover, while the events are predicted with acceptable levels of precision, the recall is much lower, especially in full-texts.

#### 4.2.3. Negation and Speculation Detection

Table 7 shows the best-performing systems in Task 3, corresponding to the identification of negations and speculations. In the second edition only two teams participated in this task, both presenting an important improvement over the best result of 2009 (ConcordU09 [84]), with UTurku [64, 77] showing a better performance in extracting negated events, and ConcordU11 [93] showing a better performance in extracting speculated events and better overall results in terms of full-texts. As can be directly seen from lower precision and recall rates achieved, this task is considerably more difficult than the extraction of secondary arguments. Although the dataset is different, preventing direct comparison, the results achieved for full-texts on the last edition of the task were similar to the previous results.

## 5. Discussion and Future Research Directions

Biomolecular event extraction consists of identifying alterations in the state of a biomolecule or interactions between two or more biomolecules, described in natural language text in the scientific literature. These events constitute the building blocks of biological processes and functions, and automatically mining their descriptions has the potential of providing insights for the understanding of physiological and pathogenesis mechanisms. Event extraction has been addressed through multiple approaches, starting from basic pattern matching and parsing techniques to machine learning methods.

Despite the steady progress shown over the last decade, the current state-of-the-art performance clearly shows that extracting events from biomedical literature still presents various challenges. While performance results close to 80% in *F*-score have been achieved in the recognition of simpler events, the extraction of more complex events such as binding and regulation events is still limited. Although substantial efforts have been made for the recognition of these events, the best performance achieved remains 30%–40% lower than that for simple events.

### 5.1. Patterns and Matching Rules versus Machine Learning-Based Approaches

Biomedical event extraction has been moving from purely rule-based and dictionary-based approaches towards ML-based solutions, due to the difficulty in creating sufficiently rich rules that capture the variability and ambiguity of natural language, leading to limited generalization capability and lower recall. Nonetheless, the automatic extraction of rules from annotated data may help in obtaining richer rules. In the third edition of the BioNLP-ST, for instance, the rule-based BioSEM system presented significantly higher precision than the best ML approaches, although with a lower recall.

On the other hand, and despite showing the best performance results in a shared task setting, machine learning approaches present important drawbacks, namely, their dependence on sufficiently large and high-quality training datasets. Another important limitation is that even if such a dataset exists, as in the case of evaluation tasks, its focus may be too restricted which could mean that a model trained on these data would be well tuned for extracting information from similar documents but could become unusable in a slightly different domain. Many recent advances in this task have come from the combination of different systems and approaches. For example, rule-based systems have been applied to derive constrains from the manually annotated data that are then used to correct or filter the results of the machine learning-based event extraction. Another option is to combine the results of rule-based and ML-based methods in an ensemble approach.

### 5.2. Feature Selection and Feature Reduction

The feature extraction process generates a wide range of features of different nature. In many studies, the generation of the final data representation consists of extracting as many features as possible and integrating them in a basic way. This produces a high dimensional space that does not take into account multiple aspects regarding the nature of the data, such as redundancy, noisy information, or the complexity of its representation space. Although some studies have tried to address this problem, this has mainly been from the point of view of reducing the dimensionality. Some works have shown that an analysis of the contribution of features and appropriate selection of these can significantly reduce the computational requirements. For instance, Campos et al. [42] proposed a solution that chooses the features that better reflect the linguistic characteristics of the triggers for a particular event type; these features are automatically selected via an optimization problem. Also, Van Landeghem et al. [74] showed that a similar overall performance could be achieved using less than 50% of the originally extracted features. Another important consideration is that this reduction not only avoids extra processing time but also helps to avoid undesirable noise [92].

### 5.3. Current Trends and Challenges

Most event extraction strategies split the problem into two main steps: a first step consisting of the identification of trigger words that indicate the events and a second step (edge detection) that fully specifies the events by adding the corresponding arguments. This makes trigger word detection a crucial task in event extraction, since the second step is commonly performed over the results of that process. In fact, some studies have shown that missing triggers cause about 70% of all errors in event detection [89]. To address these cascading errors, some authors have proposed the joint prediction of triggers and edges connecting these triggers to participants in the event [101, 102, 104, 106, 112]. As shown by the comparative results, this joint inference allowed the most significant advances in terms of prediction performance and constitutes the state-of-the-art approach for event detection. Structured prediction and jointly trained models have also been applied successfully in other biomedical information extraction tasks. Berant et al. [113], for example, used event extraction in order to improve fine-grained information extraction for question answering, applying the structured averaged perceptron algorithm to jointly extract the event triggers and arguments. Kordjamshidi et al. [114] applied structured prediction to the task of extracting information on bacteria and their locations (e.g., host organism) by jointly identifying mentions of entities, organisms, and habitats and corresponding localization relationship. They used a set of local and contextual features for words and phrases and for pairs of phrases and trained structured SVMs for jointly extracting the information.

The use of postprocessing rules to filter and refine the results of model predictions has proved to be an essential step in event extraction. These rules are usually automatically obtained from annotated data and reflect restrictions or likelihoods for the creation of edges between triggers and participants in the construction of the events. On the other hand, the application of automatically extracted rules, on their own, has also shown positive results as shown by the BioSEM system. The ensemble combination of this strategy with the results from ML models could provide a way of balancing the precision and recall of each approach.

While the initial efforts in this task focused on the analysis of abstracts, this greatly limits the amount of information that can be extracted and therefore the impact of these methods on downstream applications, such as question answering, network construction and curation, or knowledge discovery. The latest attempts have therefore focused on mining full-text documents but, as expected, the precision of event extraction using the full body is lower due to the more complex language used in the main text of the publications. Interestingly, the results obtained have shown that while the recognition of complex events becomes more difficult in full-texts, the recognition performance for simple events is higher.

Improving the extraction of complex events, namely, from full-text documents, either through rules, ML, or hybrid approaches, may depend on the amount and quality of the training data. However, the construction of a fully annotated large-scale dataset that covers the wide variety of linguistic patterns would be a very demanding and unfeasible task. To overcome this, repositories with large amounts of nonannotated data, such as PubMed, could be exploited by unsupervised and semisupervised machine learning methods, to construct richer text representations that can better model complex relations between words. This is a very promising research direction due to the large amount of available data [1] but, unfortunately, very few studies try to take advantage of this unstructured information (i.e., raw text without annotations). Another interesting aspect that could also be further explored is the incorporation of domain information in resources such as dictionaries, thesaurus, and ontologies. Related concepts and semantic relations obtained from these resources could be used to enrich the representation of textual instances or to aid in the generation of filtering and postprocessing rules.

Another major challenge for event extraction is related to coreferences and anaphoric expressions, which make the correct identification of event participants more difficult. This is a very active research field in computational linguistics and natural language processing and has also been vastly studied in the specific case of biomedical text mining [75, 115, 116]. The second edition of the BioNLP-ST included coreference resolution as a supporting task, in which the best participants obtained results ranging from 55% to 73% in precision, for a recall varying between 19% and 22%. These results show that there is still much room for improvement in this area, which would also enhance the event extraction results.

Additionally to the extraction of events, respective types, and participants, a more complete specification of events requires the identification of additional arguments, such as specific binding sites, protein regions, or domains. This extraction of fine-grained information is inherently more difficult than the primary identification of events, as can be seen from the current state-of-the-art performance. However, this information is required if the automatically extracted events are to be used for constructing biological networks [2]. Similarly, the identification of negation and speculation, also addressed by various works and evaluated in the BioNLP-ST setting, still represents a very difficult challenge. Nonetheless, even if current limitations still hinder the direct extraction of reliable biological networks from scientific texts, the existing methods can serve as an efficient aid to accelerate the process of network extraction, when integrated in curation pipelines that allow simple and user-friendly revision, correction, and completion of the extracted information.

## 6. Conclusions

This paper presents a review of the state-of-the-art in biomolecular event extraction, which is a challenging task due to the ambiguity and variability of scientific documents, and the complexity of the biological processes described. Over the last decades a wide range of approaches have been proposed, ranging from basic pattern matching and parsing techniques to sophisticated machine learning methods.

Current state-of-the-art methods use a stacked combination of models, in which the second model either uses rules to refine the initial predictions or applies reranking to select the best event structures. Additionally, the joint prediction of the full event structure as opposed to a two- or three-stage approach has shown to produce improved results.

Important challenges still exist, namely, in the extraction of complex regulation events, in the resolution of coreferences, and in the identification of negation and speculation. Nonetheless, current methods can be used in text-mining-assisted curation pipelines, for network construction and population of knowledge bases.

## Figures and Tables

**Table 1:** Most common features used in the main event detection stages.

Feature groups	Features	Trigger recognition	Edge detection
Token	Part-of-speech	**X**	**X**
	Lemma		
	Orthographic	**X**	
	Char n-grams	**X**	
	Word shape	**X**	
	Prefixes/suffixes	**X**	

Sentence and local context	Number of entities	**X**	
	BoW counts	**X**	
	Windows or conjunctions of features	**X**	

Dependency	Number and type of dependency edges	**X**	
	Words, lemmas, or POS tags in dependency path	**X**	**X**
	N-grams in dependency path	**X**	**X**

External resources	WordNet lemmas	**X**	**X**
	Trigger lexicon	**X**	**X**
	Entity lexicon	**X**	**X**

**Table 2:** Most relevant work addressing the problem of trigger detection. Studies are listed in chronological order and the different approaches are classified in three main groups: rule-based, dictionary-based, and ML-based strategies.

Approach						Reference
Rule-based	Dictionary-based	ML-based				
		SVM	CRF	VSM	MEMM	
X	X					Kilicoglu and Bergler 2009 [84]
	X		X			MacKinlay et al. 2009 [85]
		X (structural)	X			Björne et al. 2009 [86]
	X					Miwa et al. 2010 [87]
X	X					Le Minh et al. 2011 [70]
X	X	X				Kilicoglu and Bergler 2011 [79]
X						Casillas et al. 2011 [88]
X		X (L, R)				Van Landeghem et al. 2011 [74]
		X (P)	X	X (CS)		Martinez and Baldwin 2011 [73]
					X	Zhou and He 2011 [89]
		X (L)				Miwa et al. 2012 [75]
		X (L)				Björne et al. 2012 [64]
		X (C)				Qian and Zhou 2012 [90]
		X (L)				Wang et al. 2013 [65]
		X (L)				Hakala et al. 2013 [91]
		X (L)				Zhang et al. 2013 [43]
		X (L)				Liu et al. 2013 [72]
			X			Campos et al. 2014 [42]
		X (L)				Xia et al. 2014 [92]

L: linear kernel; R: radial basis function kernel; P: polynomial kernel; C: convolution tree kernel; CS: cosine similarity.

**Table 3:** Most relevant work addressing the problem of edge detection. Studies are listed in chronological order and the different approaches are classified in three main groups: rule-based, dictionary-based, and ML-based strategies.

Approach					Reference
Rule-based	Dictionary-based	ML-based			
		SVM	CRF	HVS	
**X**	**X**				Kilicoglu and Bergler 2009 [84]
**X**		**X**			Björne et al. 2009 [86]
**X**					MacKinlay et al. 2009 [85]
		**X** (L)			Miwa et al. 2010 [87]
**X**					Le Minh et al. 2011 [70]
**X**					Kilicoglu and Bergler 2011 [79]
		**X** (L)		**X**	Zhou and He 2011 [89]
			**X**		Martinez and Baldwin 2011 [73]
		**X** (L)			Miwa et al. 2012 [75]
		**X** (L)			Björne et al. 2012 [64]
		**X** (L)			Wang et al. 2013 [65]
		**X** (L)			Hakala et al. 2013 [91]
		**X** (L)			Xia et al. 2014 [92]

L: linear kernel.

**Table 4:** Modality detection. Most relevant work addressing the problem of modality detection classified in rule-based, dictionary-based, and ML-based strategies.

Approach				Reference
Rule-based	Dictionary-based	ML-based		
		SVM	CRF	
**X**	**X**			Kilicoglu and Bergler 2009 [84]
		**X** (L)		Björne et al. 2009 [86]
			**X**	MacKinlay et al. 2009 [85]
		**X** (L)		Miwa et al. 2010 [87]
	**X**			Kilicoglu and Bergler 2011 [79]
		**X** (L)		Miwa et al. 2012 [75]
		**X** (L)		Björne et al. 2012 [64]
		**X** (L)		Van Landeghem et al. 2013 [110]
		**X** (L)		Xia et al. 2014 [92]

L: linear kernel.

**Table 5:** Core event extraction performance comparison. BioNLP shared task comparison results in recall/precision/*F*-score (%) on the test set for Task 1 (core event extraction). (A) abstracts only and (F) full papers. Data extracted from BioNLP-ST 2009, BioNLP-ST 2011, and BioNLP-ST 2013 overviews [51, 52, 109].

Year	System		Event type			Total
			Simple	Binding	Regulation	
2009	UTurku Björne et al. [86]	(A)	64.21/77.45/70.21	40.06/49.82/44.41	35.63/45.87/40.11	46.73/58.48/51.95

2010	Miwa Miwa et al. [87]	(A)	65.31/76.44/70.44	52.16/53.08/52.62	35.93/46.66/40.60	48.62/58.96/53.29

2011	FAUST Riedel et al. [111]	(A) (F)	66.16/81.04/72.85 75.58/78.23/76.88	45.53/58.09/51.05 40.97/44.70/42.75	39.38/58.18/46.97 34.99/48.24/40.56	50.00/67.53/57.46 47.92/58.47/52.67
	UMass Riedel and McCallum [104]	(A) (F)	64.21/80.74/71.54 75.58/83.14/79.18	43.52/60.89/50.76 41.67/47.62/44.44	38.78/55.07/45.51 34.72/47.51/40.12	48.74/65.94/56.05 47.84/59.76/53.14

2013	EVEX Hakala et al. [91]	(F)	73.83/79.56/76.59	41.14/44.77/42.88	32.41/47.16/38.41	45.44/58.03/50.97
	TEES-2.1 Björne and Salakoski [97]	(F)	74.19/79.64/76.82	42.34/44.34/43.32	33.08/44.78/38.05	46.17/56.32/50.74
	BioSEM Bui et al. [99]	(F)	67.71/86.90/76.11	47.45/52.32/49.76	28.19/49.06/35.80	42.47/62.83/50.68

**Table 6:** Event enrichment performance comparison. BioNLP shared task comparison results in recall/precision/*F*-score (%) on the test set for Task 2 (event enrichment). (A) abstracts only and (F) full papers. Data extracted from BioNLP-ST 2009, BioNLP-ST 2011, and BioNLP-ST 2013 overviews [51, 52, 109].

Year	System		Site	Localization	Total
2009^a^	UTurku + DBCLS09 Björne et al. [86]	(A)	71.43/71.43/71.43	23.08/88.24/36.59	32.14/72.41/44.52

2011^b^	FAUST Riedel et al. [111]	(A) (F)	43.51/71.25/54.03 17.58/69.57/28.07	36.92/77.42/50.00 —	41.33/72.97/52.77 17.39/66.67/27.59
	UMass Riedel and McCallum (b) [104]	(A) (F)	42.75/70.00/53.08 16.48/75.00/27.03	36.92/77.42/50.00 —	40.82/72.07/52.12 16.30/75.00/26.79

2013^c^	TEES-2.1 Björne and Salakoski [97]	(F)	20.68/59.82/30.73	36.67/78.57/50.00	22.03/61.90/32.50
	EVEX Hakala et al. [91]	(F)	19.44/59.43/29.30	36.67/78.57/50.00	20.90/61.67/31.22

^a^Only phosphorylation sites were considered.

^b^The results are for overall binding and phosphorylation sites.

^c^The task included the prediction of sites for other protein modification and regulation events.

**Table 7:** Negation and speculation detection performance comparison. BioNLP shared task comparison results in recall/precision/*F*-score (%) on the test set for Task 3 (negation/speculation detection). (A) abstracts only and (F) full papers only. Data extracted from BioNLP-ST 2009, BioNLP-ST 2011, and BioNLP-ST 2013 overviews [51, 52, 109].

Year	System		Negation	Speculation	Total
2009	ConcordU09 Kilicoglu and Bergler [84]	(A)	14.98/50.75/23.13	16.83/50.72/25.27	15.86/50.74/24.17

2011	UTurku Björne et al. [64, 77]	(A) (F)	22.03/49.02/30.40 25.76/48.28/33.59	19.23/38.46/25.64 15.00/23.08/18.18	20.69/43.69/28.08 19.28/30.85/23.73
	ConcordU11 Kilicoglu and Bergler [93]	(A) (F)	18.06/46.59/26.03 21.21/38.24/27.29	23.08/40.00/29.27 17.00/34.69/22.82	20.46/42.79/27.68 18.67/36.14/24.63

2013	TEES-2.1 Björne and Salakoski [97]	(F)	21.68/36.84/27.30	18.46/33.96/23.92	19.53/35.59/25.22
	EVEX Hakala et al. [91]	(F)	20.98/38.03/27.04	18.46/32.73/23.61	19.82/34.41/25.15
